# Nonlinear Associations of Uric Acid and Mitochondrial DNA with Mortality in Critically Ill Patients

**DOI:** 10.3390/jcm14134455

**Published:** 2025-06-23

**Authors:** Max Lenz, Robert Zilberszac, Christian Hengstenberg, Johann Wojta, Bernhard Richter, Gottfried Heinz, Konstantin A. Krychtiuk, Walter S. Speidl

**Affiliations:** 1 Department of Internal Medicine II, Division of Cardiology, Medical University of Vienna, 1090 Vienna, Austria; max.lenz@meduniwien.ac.at (M.L.); robert.zilberszac@meduniwien.ac.at (R.Z.); christian.hengstenberg@meduniwien.ac.at (C.H.); johann.wojta@meduniwien.ac.at (J.W.); bernhard.richter@meduniwien.ac.at (B.R.); gottfried.heinz@meduniwien.ac.at (G.H.); walter.speidl@meduniwien.ac.at (W.S.S.); 2 Ludwig Boltzmann Institute for Cardiovascular Research, 1090 Vienna, Austria; 3 Core Facilities, Medical University of Vienna, 1090 Vienna, Austria

**Keywords:** critical care, uric acid, mtDNA, survival, nonlinear correlation

## Abstract

**Background**: Mitochondrial DNA (mtDNA) has strong pro-inflammatory potential and was found to be associated with mortality in critically ill patients. The purine bases from circulating cell-free DNA, including mtDNA, are catabolised into uric acid, contributing to elevated systemic levels. However, the prognostic value of uric acid in unselected critically ill intensive care unit (ICU) patients remains unclear. We aimed to investigate the association between uric acid levels at admission and 30-day mortality, assess its correlation with mtDNA, and examine prognostic relevance based on the primary cause of admission. **Methods**: This prospective single-centre study included 226 patients admitted to a tertiary care ICU. Uric acid and mtDNA levels were assessed at admission. Survival analyses were performed in the overall cohort and in subgroups stratified by primary diagnosis. **Results**: Uric acid showed a U-shaped association with 30-day mortality, with both low and high levels linked to reduced survival. In multivariate analysis, the 4th quartile of uric acid remained associated with adverse outcomes, independent of sex, vasopressors, mechanical ventilation, and creatinine (HR 2.549, 95% CI: 1.310–4.958, *p* = 0.006). A modest correlation was observed between uric acid and mtDNA (r = 0.214, *p* = 0.020). However, prognostic relevance varied by diagnosis. While uric acid predicted mortality in patients following cardiac arrest (*p* = 0.017), mtDNA was found to bear prognostic value in cardiogenic shock and decompensated heart failure (*p* = 0.009). **Conclusions**: Uric acid was independently associated with mortality in critically ill patients, with both low and high levels carrying prognostic value. Its predictive capabilities differed from mtDNA but showed partial overlap. However, both markers exhibited varying prognostic performance depending on the primary cause of admission.

## 1. Introduction

Although patients admitted to the intensive care unit (ICU) present with a variety of underlying conditions, systemic immune dysregulation with pronounced inflammation remains a hallmark of critical illness. Importantly, distinguishing between infectious and sterile inflammation remains challenging and continues to complicate clinical management [1]. Tissue injury and associated cell death facilitate the release of damage-associated molecular patterns (DAMPs). As they are recognised via pattern recognition receptors, a prompt immune response is mounted by the innate immune system [2]. Interestingly, both pathogen-associated and damage-associated activations are mediated through the same pattern recognition receptors, including the Toll-like receptor (TLR) family, and subsequent cellular downstream signalling. This might account for the observed similarity between various hyperinflammatory pathologies encountered at the ICU [3].

Among various DAMPs, mitochondrial DNA (mtDNA) was found to have high prognostic value for the survival of critically ill patients [4,5]. Its pro-inflammatory potential is thought to relate to the endosymbiont theory, which states that mitochondria evolved from bacteria and retain certain features recognised by TLRs [6]. In 2019, Harrington et al. published a systematic review of 40 studies encompassing over 3400 critically ill patients. They confirmed a consistent association between circulating mtDNA levels and mortality, with AUROC (area under the receiver operating characteristic) values ranging from 0.61 to 0.95. Moreover, the authors emphasised substantial methodological heterogeneity and highlighted the need for standardised protocols to utilise mtDNA as a biomarker [7]. The limited feasibility of mtDNA measurement includes non-standardised extraction protocols, variable primer design, and inconsistent sample processing. Despite growing evidence from our group and others, this methodological variability may explain why mtDNA has not yet found broader application in clinical risk stratification.

Uric acid may serve as a surrogate for mtDNA in the ICU setting and is more readily available in clinical practice. Cellular damage leads to the release of cell-free DNA, including both nuclear and mitochondrial DNA, into the circulation. Upon degradation, purine bases are catabolised into uric acid, contributing to elevated systemic levels. As a byproduct of purine metabolism, uric acid is often elevated in critically ill patients and may reflect systemic injury and inflammation, particularly in conditions such as sepsis or acute kidney injury [8,9,10]. Therefore, we aimed to highlight the prognostic relevance of uric acid and explore its potential role as a surrogate marker for mtDNA. Data from the emergency department and preoperative settings suggest that uric acid is associated with patient outcomes and subsequent renal function [11,12,13]. Surprisingly, adequately powered studies regarding the prognostic value in unselected medical patients appear to be lacking. Therefore, a primary and two secondary research questions were formulated: Firstly, does uric acid predict 30-day survival in unselected critically ill patients? Secondly, is there a correlation between uric acid and mtDNA, and are there differences in uric acid versus mtDNA when stratified for the primary cause of admission? A total of 226 patients admitted to a tertiary ICU were analysed to provide further insight into these pending questions.

## 2. Materials and Methods

### 2.1. Design and Study Population

This study was designed as a prospective observational study conducted at the tertiary care medical ICU of the Division of Cardiology, Department of Internal Medicine II at the Vienna General Hospital. Patients treated at our ICU exhibit a wide spectrum of critical illness. Nevertheless, our expertise lies in the treatment of acute cardiovascular conditions. Therefore, the included population comprises a high number of patients admitted following cardiac arrest or due to cardiogenic shock, acute decompensated heart failure, and cardiac surgery. Our goal was to collect an unselected study cohort of critically ill patients; therefore, we enrolled all consecutive individuals admitted over one year. All conscious individuals were included after providing written informed consent [14]. In case of unconsciousness, the need for informed consent was waived by the ethics committee. The study included patients aged 18 years and older. Individuals with active human immunodeficiency virus (HIV) or hepatitis C virus (HCV) infections were excluded for safety reasons. The study was conducted in accordance with the Declaration of Helsinki and the International Conference on Harmonisation (ICH) Good Clinical Practice guidelines, which were adhered to during both the planning and execution phases. The study protocol was approved by the local ethics committee of the Medical University of Vienna (EK 1101/2012), and a total of 233 patients were enrolled. Uric acid values were available for 226 patients comprising the analysed dataset. The primary study endpoint was defined as 30-day mortality. All included patients were followed up for 30 days or until the endpoint was reached.

### 2.2. Data Acquisition and Blood Sampling

Baseline characteristics, including the primary diagnosis, medical history, and routine laboratory parameters, were documented within the first 24 h following ICU admission. In addition, major interventions performed prior to, or within the first 72 h of the stay, were recorded and included mechanical ventilation, renal replacement therapy, major surgical procedures, administration of catecholamines, and extracorporeal membrane oxygenation (ECMO). Disease severity and associated mortality risk were determined using the Simplified Acute Physiology Score II (SAPS II), the Acute Physiology and Chronic Health Evaluation II (APACHE II) score, and the Sequential Organ Failure Assessment (SOFA) score. Blood samples were obtained at ICU admission, preferably via an arterial line. If no arterial line was available, central venous access was used. To maintain sample integrity, the initial 3 mL of blood was discarded. Laboratory measurements were conducted through the Department of Laboratory Medicine of the Medical University of Vienna. Additionally, biobanking involved the collection of one EDTA tube, one 3.8% sodium citrate tube, and one serum separator tube (Greiner Bio-One, Kremsmünster, Austria). These were centrifuged at 4 °C (3000 RPM, 15 min) and stored at −80 °C [4,15].

### 2.3. Measurement of mtDNA

Preparation and measurement of mtDNA were done as previously described [4]. In short, DNA from platelet-poor plasma fraction was extracted using Qiagen DNeasy Blood Mini Kit (Qiagen, Venlo, The Netherlands) on an automated QIAcube platform (Qiagen). Using LightCycler TaqMan Master (Roche, Basel, Switzerland), DNA copy numbers were quantified by real-time polymerase chain reaction targeting the mitochondrial genes cytochrome b, cytochrome c oxidase subunit III, and nicotinamide adenine dinucleotide dehydrogenase. Cycling conditions were chosen as initial denaturation at 95 °C for 10 min, followed by 60 cycles of 95 °C for 15 s and 60 °C for 30 s. Primers were designed via the Roche Universal Probe Library Assay Design Centre (https://primers.neoformit.com/, accessed on 20 June 2025). A mitochondria isolation kit (Pierce, Thermo Fisher Scientific, Waltham, MA, USA) was used to isolate mitochondria from human smooth muscle cells and establish a standard curve for mtDNA quantification. The mean of all three gene targets was used to calculate circulating plasma mtDNA levels.

### 2.4. Statistical Analysis

Sample size calculation indicated that, in a cohort with a mortality rate of 25% (based on mortality numbers in our ICU of the previous three years), a total of 200 patients would be required to detect a 50% difference in uric acid or mtDNA plasma levels between survivors and non-survivors, assuming a power of 0.8 and a significance level of 0.01. Continuous variables are depicted as median and interquartile range (IQR), and Levene’s test was used to assess equality of variances. For categorical variables, counts or percentages were provided and compared using either the Chi-square or Fisher’s exact test, as appropriate. Non-parametric data were analysed using the Mann–Whitney U test, while the unpaired Student’s *t*-test was used for parametric data. The Kolmogorov–Smirnov test was applied to distinguish between parametric and non-parametric data. While normality was tested, continuous variables were uniformly reported as median and IQR to maintain consistency in data presentation. Kruskal–Wallis test with Dunn’s post hoc testing (Bonferroni corrected) was used for the comparison of multiple groups. Kaplan–Meier curves illustrated the predictive properties of quartiles of uric acid (individually as well as 1st to 3rd quartiles versus the 4th quartile) regarding 30-day mortality. Levels of significance were determined by the log-rank test. Furthermore, Cox-proportional hazard regression analysis was conducted in a model adjusting for sex, use of vasopressors, mechanical ventilation, and creatinine. Receiver operating characteristic (ROC) curves were generated for 30-day survival using uric acid and mtDNA as test variables. To investigate potential nonlinear associations of uric acid and mtDNA levels with survival, quadratic logistic regression models were applied. Both linear and squared terms were entered into the models to assess inverted U-shaped or threshold relationships. Polynomial contrast analysis (linear and quadratic components) was conducted to test for trend shapes across quartiles formally. For statistical analysis, SPSS Statistics 28.0 (IBM Corporation, New York, NY, USA) and R (version 4.2.3, the R Foundation for Statistical Computing, Vienna, Austria) were utilised. While a significance level of *p* = 0.01 was used for the sample size calculation to ensure adequate power, a threshold of *p* < 0.05 was applied for hypothesis testing throughout the study.

## 3. Results

Of the 233 initially selected patients, 226 had available values of uric acid and were therefore included. Demographics and baseline characteristics can be found in Table 1 and are clustered according to 30-day survival. The overall mortality rate of 25.7% (58 out of 226 patients) constitutes a severely ill patient collective, underlined by a high APACHE II (20.0, IQR 12.8–25.0) as well as SAPS II score (45.0, IQR 31–59). The median age within the observed population was 66.3 years (IQR 55.0–76.3), and 38.5% of patients were female. Several factors were found to be significantly different in survivors versus non-survivors. These included the use of mechanical ventilation (*p* = 0.003) and vasopressors (*p* < 0.001) as well as the initial measurements of creatinine (*p* < 0.001), arterial pH (*p* = 0.002), and lactate (*p* = 0.001, all Table 1). The most frequent primary cause of admission was found to be cardiogenic shock and acute decompensated heart failure (23.0%), followed by cardiac arrest (22.6%) and post-operative observation after cardiac surgery (20.8%).

**Figure 1:**
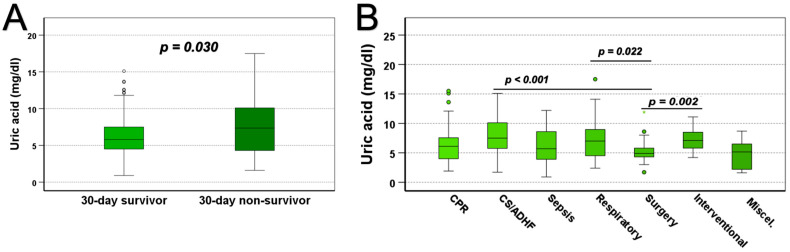
Levels of uric acid in 30-day survivors versus non-survivors are displayed in (**A**) (n = 168/58 tested by the Mann–Whitney U test). Stratification for the primary cause of admission (n = 51/52/22/23/47/25/6) highlights differences between cardiac (CS/ADHF), respiratory, interventional, and surgical subgroups in (**B**) (Tested by Kruskal–Wallis test with Dunn’s post hoc testing—Bonferroni corrected). A total of 226 patients were analysed, and *p*-values of <0.05 are considered statistically significant. CPR: cardiopulmonary resuscitation; CS: cardiogenic shock; ADHF: acute decompensated heart failure.

Within the total study population, uric acid levels were found to be significantly elevated in 30-day non-survivors (Figure 1A, *p* = 0.030). Stratifying for the primary cause of admission, Kruskal–Wallis test with Dunn’s post hoc testing revealed tangible differences (*p* < 0.001, Figure 1B). Moreover, patients admitted due to cardiogenic shock and acute decompensated heart failure (cardiac, Figure 1B, *p* < 0.001) as well as those suffering from respiratory failure (respiratory, Figure 1B, *p* = 0.022) and those undergoing minimal invasive valve interventions (interventional, Figure 1B, *p* = 0.002) exhibited higher levels of uric acid compared to individuals admitted for post-operative observation after cardiac surgery (surgery, Figure 1B).

**Figure 2:**
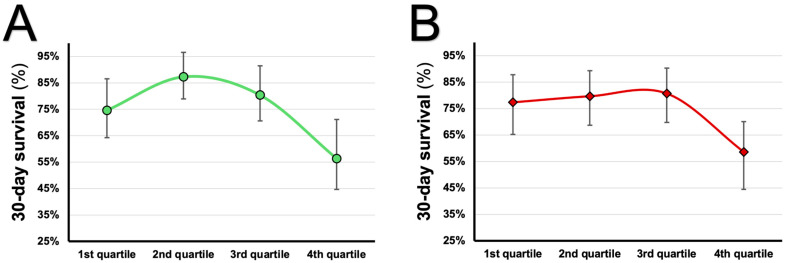
(**A**,**B**) show a nonlinear distribution of survival in quartiles of uric acid (green, (**A**)) and mtDNA (red, (**B**)) based on 30-day survival ± 95% confidence interval. A total of 226 patients were analysed, and *p*-values of <0.05 are considered statistically significant.

A significant correlation was found between uric acid and mtDNA (r = 0.214, *p* = 0.020, Supplemental Figure S1). However, given the weak correlation, any interpretation should be made cautiously [16]. Furthermore, a visual representation of the 30-day survival is depicted for quartiles of uric acid and mtDNA. Both parameters exhibit a nonlinear relationship with the average 30-day survival. When stratified by uric acid quartiles, survival peaked at the 2nd and 3rd, followed by a marked decline at the 4th quartile (Figure 2A). Chi-square analysis confirmed a significant association between uric acid quartiles and survival (Pearson’s Chi-square *p* = 0.002). The survival pattern was visually compatible with an inverted U-shaped relationship (U-shaped in terms of mortality). However, formal quadratic trend testing did not reach statistical significance (*p* = 0.093). In contrast, mtDNA showed a significant association with survival across quartiles (Pearson’s Chi-square *p* = 0.032) and a significant quadratic trend (*p* = 0.044), driven by a decline in survival at the highest quartile (Figure 2B). This pattern indicates a threshold effect rather than a classical U- or J-shaped relationship, with survival remaining stable across lower quartiles but decreasing notably at the highest mtDNA levels.

**Figure 3:**
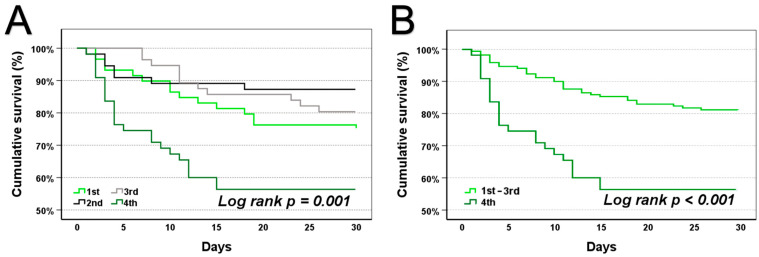
Kaplan–Meier analyses show a significant difference in cumulative survival individually (**A**) as well as comparing the 1st to 3rd quartiles to the 4th quartile of uric acid (**B**). A total of 226 patients were analysed, and *p*-values of <0.05 are considered statistically significant.

Additionally, Kaplan–Meier analyses were used to assess the prognostic value of uric acid. First, stratification into quartiles revealed the highest cumulative mortalities in the 1st and 4th quartiles. Furthermore, the first to third quartiles were combined and compared to the fourth quartile. Both depicted Kaplan–Meier analyses displayed significant differences in cumulative survival for uric acid (Figure 3A,B, log-rank *p* = 0.001 and *p* < 0.001).

Moreover, cox-proportional hazard regression analysis revealed significant prognostic value of uric acid measurements at admission regarding 30-day mortality. A univariate analysis comparing the first to the fourth quartile demonstrated a hazard ratio (HR) of 2.067 (Table 2, 95% confidence interval (CI) 1.084–3.944, *p* = 0.027). In a corresponding multivariate analysis, these results were proven independent of sex, vasopressors, mechanical ventilation, and serum creatinine with an HR of 2.549 (Table 2, 95% CI 1.310–4.958, *p* < 0.006).

**Figure 4:**
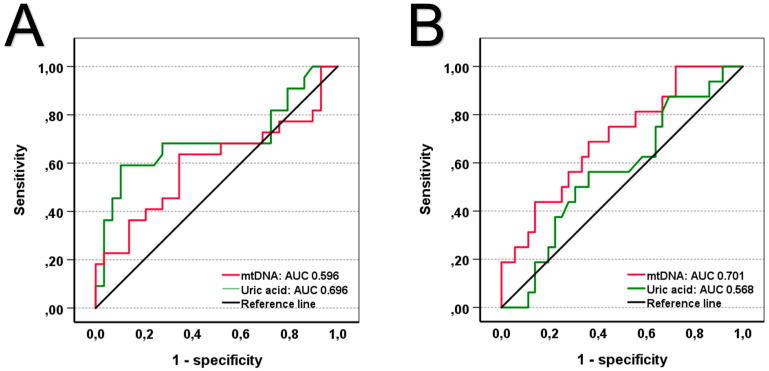
ROC curves predicting 30-day mortality in patients admitted after CPR (**A**) and in patients suffering from cardiogenic shock or acute decompensated heart failure (**B**). A total of 103 patients were included in these analyses (CPR, n = 51; CS/ADHF, n = 52). *p*-values of <0.05 are considered statistically significant. mtDNA: mitochondrial DNA; AUC: area under the curve; CPR: cardiopulmonary resuscitation; CS: cardiogenic shock; ADHF: acute decompensated heart failure.

Finally, subgroup analyses for the primary cause of admission found differences in the prediction of 30-day mortality comparing uric acid and mtDNA. The 30-day non-survivors admitted following cardiac arrest had significantly higher levels of uric acid but not mtDNA compared to survivors (Supplementary Table S1). On the other hand, patients admitted due to cardiogenic shock or acute decompensated heart failure displayed significantly elevated levels of mtDNA but no differences in uric acid (Supplementary Table S1). Moreover, differences in positive prediction rates are present when comparing uric acid and mtDNA. ROC curves predicting 30-day mortality revealed an AUC of 0.696 for uric acid and 0.596 for mtDNA in patients admitted following cardiac arrest (Figure 4A). In contrast, patients admitted with cardiogenic shock or acute decompensated heart failure showed a different pattern, with an AUC of 0.701 for mtDNA and 0.568 for uric acid (Figure 4B). While these values reflect only moderate discrimination, they may still offer insight into context-specific prognostic performance. Predictive properties of uric acid and mtDNA for the remaining causes of admission can be found in Supplementary Table S1.

## 4. Discussion

Previous studies report hyperuricemia to be associated with increased 90-day mortality in ICU patients with sepsis [8]. Similarly, elevated uric acid levels have been linked to mortality in sepsis-associated acute kidney injury and ARDS [9,17,18]. While existing evidence suggests a prognostic role for uric acid in selected pathologies and emergency settings, adequately powered studies in unselected critically ill populations remain scarce [13,19]. Nevertheless, while hyperuricemia may indicate acute cellular damage or oxidative stress, it is not limited to critical illness. Changes in circulating plasma levels may reflect preexisting conditions such as chronic kidney disease, metabolic disorders, high cell turnover, or use of specific drugs [7]. Our findings demonstrate a significant association between uric acid and 30-day mortality in a heterogeneous ICU cohort. This relationship was confirmed in both univariate and multivariate Cox regression models and remained independent of sex, vasopressor use, mechanical ventilation, and creatinine levels. Notably, survival analysis across uric acid quartiles revealed an inverted U-shaped distribution, with reduced survival at both the lowest and highest quartiles corresponding to a U-shaped distribution in terms of mortality. Interestingly, a similar U-shaped distribution regarding all-cause mortality was reported in 375.163 patients undergoing health check-ups [20]. Tseng et al. also reported yet another U-shaped association between serum uric acid levels and cardiovascular mortality, discussing the role of malnourishment in this setting [21]. Taken together, these observations support the idea that both low and high uric acid levels reflect distinct but unfavourable physiological states. Hypouricemia may point to liver dysfunction, malnutrition, or dilutional effects, while hyperuricemia may result from purine overload, oxidative stress, or impaired renal clearance [22]. Although pathophysiologically different, both states are linked to poor outcomes and may explain the observed U-shaped mortality patterns. Our findings highlight uric acid as a broad marker of systemic injury and metabolic stress, relevant even in unselected ICU cohorts.

Moreover, we analysed potential correlations between uric acid and mtDNA, both of which have been proposed as markers of tissue injury and mediators of systemic inflammation. While our earlier work focused on receptor-mediated pathways of mtDNA, the current study prioritises clinical feasibility and applies outcome-based quartile stratification to identify mortality patterns. Cellular damage results in the release of cell-free DNA, comprising both nuclear and mitochondrial components. As this DNA is degraded, its purine bases are metabolised into uric acid, thereby contributing to increased systemic uric acid concentrations. In this regard, uric acid may serve as a surrogate marker for mtDNA in the ICU setting, offering a more accessible parameter in routine clinical practice. In our cohort, we observed a statistically significant but rather modest positive correlation. However, the weak strength of the correlation suggests that their prognostic relevance may differ depending on the clinical context. mtDNA is known to act as a potent DAMP, triggering inflammatory responses via TLR activation in critically ill patients [4]. Uric acid, although less specific, has been linked to oxidative stress, cell injury, and immune activation [23]. The observed correlation may therefore reflect shared upstream mechanisms such as hypoxia, ischemia-reperfusion injury, apoptosis, or necrosis, all of which are associated with cell damage and DAMP release [24,25,26]. However, given the weak statistical correlation, any interpretation should be made cautiously. Moreover, survival analyses revealed distinct prognostic patterns. Uric acid showed a U-shaped association with mortality, while mtDNA demonstrated a threshold effect, with significantly reduced survival in the highest quartile only. This may indicate that uric acid reflects broader metabolic dysregulation, whereas mtDNA marks a more specific response to severe tissue injury. Given its availability, uric acid could serve as a pragmatic surrogate for generalised cell damage, whereas mtDNA may offer greater specificity in certain circumstances. Our findings provide early evidence that these two markers are partially linked and may capture complementary aspects of critical illness pathophysiology. However, the modest strength of the correlation suggests that their prognostic relevance may differ depending on the clinical context.

Finally, we analysed biomarker levels and prognostic relevance according to the primary cause of admission. Uric acid concentrations differed significantly, with the highest levels in patients admitted for cardiogenic shock or decompensated heart failure, and the lowest in those following cardiac surgery. Moreover, subgroup-specific analyses revealed divergent prognostic patterns. In patients admitted after cardiac arrest, uric acid was predictive of 30-day mortality, whereas mtDNA showed no prognostic relevance. Conversely, in patients with cardiac decompensation or cardiogenic shock, mtDNA was significantly associated with outcome, while uric acid was not. Both biomarkers demonstrated prognostic value in unselected critically ill patients, but their performance varied across clinical subgroups, which might reflect differences in underlying pathophysiologies. Cardiac arrest is characterised by global ischemia-reperfusion injury, which is known to cause the abrupt release of purines and uric acid accumulation [27]. In contrast, progressive cardiac dysfunction may result in sustained mitochondrial stress and cell turnover, with subsequent mtDNA release and associated immune activation [28,29,30]. Following this interpretation, uric acid may thus reflect acute systemic damage, while mtDNA may capture inflammatory signalling and tissue stress. Taken together, our findings support a context-dependent use of both biomarkers. This may reflect underlying pathophysiology, but given the observational design, no causal conclusions can be drawn. In line with the ROC analysis, both markers showed only moderate discriminatory performance, with AUC values around 0.70 in the best-performing subgroups. While not strong, these values may still provide context-specific prognostic information in the ICU setting and should be interpreted accordingly. Previous studies reported AUCs between 0.61 and 0.95 for mtDNA, which appears comparable to our findings and supports its moderate but context-specific prognostic utility [7]. Moreover, identifying patients with very low or high uric acid levels at admission may support early risk stratification. While causality cannot be determined, factors such as catabolism, renal clearance, or nutrition may contribute and could be targets for future studies. Moreover, whether urate-lowering therapy or nutritional adaptations are beneficial in selected patients remains a topic of discussion.

Our study has several limitations. Although uric acid and mtDNA showed prognostic value in specific subgroups, such as cardiac arrest, cardiogenic shock, and decompensated heart failure, it may be underpowered to assess predictive performance in smaller groups. The lack of an external validation cohort limits the transferability of our findings, and future multicenter studies are needed to confirm these results. While survival analysis suggested an inverted U-shaped relationship for uric acid (U-shaped for mortality), the quadratic contrast depicted a trend but did not reach statistical significance (*p* = 0.093) and should therefore be interpreted cautiously. Moreover, due to the observational design, no conclusions regarding causality can be drawn, and residual confounding cannot be excluded. However, the prospective all-comer approach strengthens the study’s applicability to routine ICU populations and reflects a broad clinical spectrum.

## 5. Conclusions

In this prospective cohort study, we assessed the prognostic relevance of uric acid and mtDNA in critically ill patients. Uric acid levels at ICU admission were significantly associated with 30-day mortality, with both low and high values linked to reduced survival. This U-shaped pattern reflects previous findings in cardiovascular mortality and large-scale population studies. We further observed a modest correlation between uric acid and mtDNA, suggesting partial overlap in their prognostic behaviour. Subgroup analyses, however, revealed differing prognostic profiles. Uric acid was predictive in patients after cardiac arrest, while mtDNA was associated with outcome in cardiogenic shock and decompensated heart failure. These findings support the use of both biomarkers for risk assessment in the ICU, while highlighting variation across clinical subgroups. Larger studies will be needed to confirm these results and better define their role in critical illness.

## Figures and Tables

**Table 1:** Baseline characteristics and survival.

		Total n = 226 (100%)	30-Day Survivors n = 168 (74.3%)	30-Day Non-Survivors n = 58 (25.7%)	*p*-Value
*** Patient characteristics:***					
	Age [years]	66.30 (55.00–76.30)	64.88 (53.16–75.79)	69.52 (59.20–77.52)	0.257
	Female sex, n (%)	87 (38.5%)	66 (39.29%)	21 (36.21%)	0.755
	Mechanical ventilation, n (%)	133 (58.85%)	89 (52.98%)	44 (75.86%)	**0.003**
	Vasopressors, n (%)	131 (58.00%)	85 (50.60%)	46 (79.31%)	**<0.001**
	C-reactive protein [mg/dL] Creatinine [mg/dL]	4.00 (1.19–10.71) 1.19 (0.88–2.06)	3.50 (0.93–10.67) 1.07 (0.86–1.80)	4.77 (1.58–13.34) 1.66 (1.16–2.81)	0.442 **<0.001**
	Bilirubin [mg/dL]	0.90 (0.52–1.50)	0.80 (0.50–1.38)	1.10 (0.60–1.80)	0.073
	Arterial pH	7.36 (7.28–7.47)	7.38 (7.30–7.47)	7.28 (7.22–7.44)	**0.002**
	Lactate [mmol/L]	1.90 (1.30–3.30)	1.8 (1.23–2.78)	3.0 (1.30–7.60)	**0.001**
	Uric acid [mg/dL]	6.10 (4.50–8.25)	5.80 (4.50–7.50)	7.20 (4.30–9.95)	**0.030**
	mtDNA [ng/mL]	21.12 (9.63–38.31)	19.79 (9.49–34.98)	26.85 (11.17–60.60)	**0.011**
	APACHE II score	20.00 (12.75–25.00)	16.50 (11.00–23.00)	27.00 (23.00–31.00)	**<0.001**
	SAPS II score	45.00 (31.00–59.00)	39.00 (29.00–50.75)	61.00 (47.00–70.00)	**<0.001**
	SOFA score	8.00 (5.00–11.00)	7.00 (5.00–10.00)	12.00 (9.00–14.00)	**<0.001**
*** Cause of admission:***					
	ADHF and cardiog. shock, n (%)	52 (23.0%)	36 (21.4%)	16 (27.6%)	0.367
	Cardiac arrest, n (%)	51 (22.6%)	29 (17.3%)	22 (37.9%)	**<0.001**
	Cardiac-surgery, n (%)	47 (20.8%)	43 (25.6%)	4 (6.9%)	**0.002**
	Valve intervention, n (%)	25 (11.0%)	24 (14.3%)	1 (1.7%)	**0.007**
	Respiratory failure, n (%)	23 (10.2%)	16 (9.5%)	7 (12.1%)	0.616
	Sepsis/septic shock, n (%)	22 (9.7%)	15 (8.9%)	7 (12.1%)	0.454
	Other reasons, n (%)	6 (2.7%)	5 (3.0%)	1 (1.7%)	1.000

Continuous values are displayed as median plus interquartile range (IQR), whereas categorical values are shown as counts and percentages. Statistically significant differences in survival are highlighted in bold numbers. A total of 226 patients were included in these analyses. Other causes of admission included bleeding (n = 2), neurologic events (n = 2), intoxication with illicit substances (n = 1), and postoperative monitoring following non-cardiac surgery (n = 1). *p*-values of <0.05 are considered statistically significant. mtDNA: mitochondrial DNA; APACHE II: Acute Physiology and Chronic Health Evaluation II; SAPS II: simplified acute physiology II; SOFA: sequential organ failure assessment; ADHF: acute decompensated heart failure.

**Table 2:** Cox regression analyses for quartiles of uric acid.

		Hazard Ratio	95% CI	*p*-Value
**Unadjusted**				**0.001**
	1st quartile of uric acid	1	-	-
	2nd quartile of uric acid	0.485	0.198–1.191	0.114
	3rd quartile of uric acid	0.732	0.336–1.593	0.431
	4th quartile of uric acid	2.067	1.084–3.944	**0.027**
**Adjusted for sex**				**0.001**
	1st quartile of uric acid	1	-	-
	2nd quartile of uric acid	0.485	0.198–1.190	0.114
	3rd quartile of uric acid	0.727	0.331–1.599	0.428
	4th quartile of uric acid	2.059	1.073–3.951	**0.030**
	Sex	1.013	0.771–1.331	0.927
**Adjusted for sex and vasopressors**				**0.002**
	1st quartile of uric acid	1	-	-
	2nd quartile of uric acid	0.592	0.240–1.464	0.257
	3rd quartile of uric acid	0.924	0.415–2.061	0.848
	4th quartile of uric acid	2.335	1.208–4.512	**0.012**
	Sex	0.951	0.721–1.256	0.725
	vasopressors	2.911	1.522–5.566	**0.001**
**Adjusted for sex, vasopressors, and mechanical ventilation**				**0.002**
	1st quartile of uric acid	1	-	-
	2nd quartile of uric acid	0.598	0.241–1.482	0.267
	3rd quartile of uric acid	0.981	0.439–2.193	0.963
	4th quartile of uric acid	2.406	1.247–4.641	**0.009**
	Sex	0.955	0.724–1.261	0.746
	Vasopressors	2.166	1.039–4.512	**0.039**
	Mechanical ventilation	1.796	0.888–3.635	0.103
**Adjusted for sex, vasopressors, mechanical ventilation, and creatinine**				**0.001**
	1st quartile of uric acid	1	-	-
	2nd quartile of uric acid	0.600	0.242–1.486	0.270
	3rd quartile of uric acid	0.990	0.442–2.215	0.980
	4th quartile of uric acid	2.549	1.310–4.958	**0.006**
	Sex	0.929	0.703–1.228	0.605
	Vasopressors	2.252	1.078–4.705	**0.031**
	Mechanical ventilation	1.758	0.867–3.567	0.118
	Creatinine	0.984	0.948–1.022	0.410

Cox regression analyses for quartiles of uric acid reveal significant predictive value in univariate as well as multivariate analyses. A total of 226 patients were included in these analyses. *p*-values of <0.05 are considered statistically significant. CI: confidence interval.

## Data Availability

The datasets used and/or analysed during the current study are available from the corresponding author upon reasonable request.

## Supplementary Materials

The following supporting information can be downloaded at: https://www.mdpi.com/article/10.3390/jcm14134455/s1, Supplemental Figure S1: Spearman rank correlation depicts the interrelationship of uric acid and mtDNA, Table S1: Subgroup analysis for uric acid and mtDNA. The graphical abstract was created in BioRender [31].
