# Supplementary material for: Nonlinear Associations of Uric Acid and Mitochondrial DNA with Mortality in Critically Ill Patients
Source: J Clin Med. 2025 Jun 23;14(13):4455. doi: 10.3390/jcm14134455 (PMC12249844; doi:10.3390/jcm14134455)
Supplement: Supplementary file 1 [file jcm-14-04455-s001.zip › jcm-3683446-supplementary.pdf]

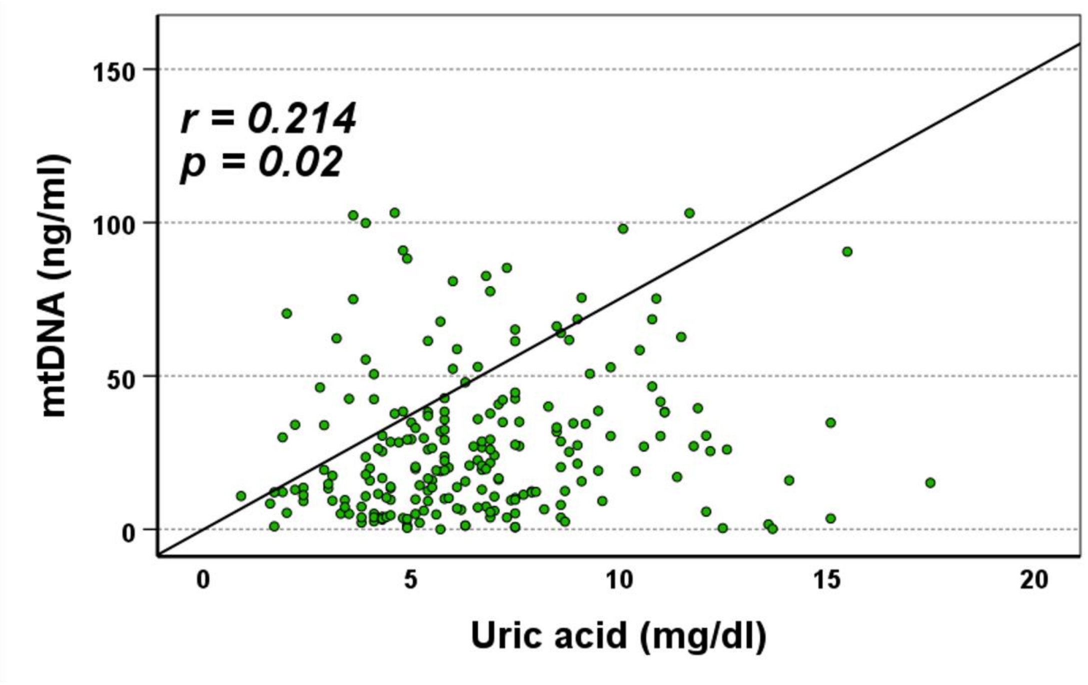

Supplemental Figure S1. Spearman rank correlation depicts the interrelationship of uric acid and mtDNA. A total of 226 patients were analysed, and p-values of < 0.05 are considered statistically significant.

Supplemental Table S1. Uric acid and mtDNA according to subgroups and 30-day survival

	Uric acid (mg/dl)	*p-value*	mtDNA (ng/ml)	*p value*
Cardiac arrest (n=51)	6.1 (3.90-7.60)	**0.017**	19.68 (9.18-38.22)	0.109
ADHF & CS (n=52)	7.5 (5.73-10.25)	0.248	29.26 (12.62-44.08)	**0.009**
Sepsis (n=22)	5.7 (3.78-8.63)	0.732	35.09 (10.83-61.68)	0.788
Respiratory (n=23)	7.0 (4.50-9.10)	0.432	19.37 (10.23-28.48)	0.660
Surgery (n=47)	4.9 (4.30-5.80)	0.856	13.33 (6.09-29.73)	0.763
Interventional (n=25)	7.1 (5.56-11.44)	0.477	22.33 (11.44-40.38)	0.415
Miscellaneous (n=6)	5.15 (2.05-7.05)	0.277	15.39 (6.93-22.38)	0.481

Supplemental Table S1. Continuous values are displayed as median plus interquartile range (IQR). Statistically significant differences in survival are highlighted in bold numbers. A total of 226 patients were included in these analyses. P-values of < 0.05 are considered statistically significant. mtDNA; mitochondrial DNA, ADHF; acute decompensated heart failure, CS; cardiogenic shock.
